# Combined features based on MT1-MMP expression, CD11b + immunocytes density and LNR predict clinical outcomes of gastric cancer

**DOI:** 10.1186/1479-5876-11-153

**Published:** 2013-06-20

**Authors:** Chun-Wei Peng, Lin-Wei Wang, Min Fang, Gui-Fang Yang, Yan Li, Dai-Wen Pang

**Affiliations:** 1 Department of Oncology, Zhongnan Hospital of Wuhan University, No. 169 Donghu Road, Wuchang District Wuhan 430071, People’s Republic China; 2 Hubei Key Laboratory of Tumor Biological Behaviors & Hubei Cancer Clinical Study Center, Zhongnan Hospital of Wuhan University, No. 169 Donghu Road, Wuchang District Wuhan 430071, People’s Republic China; 3 Department of Pathology, Zhongnan Hospital of Wuhan University, No. 169 Donghu Road, Wuchang District Wuhan, 430071, People’s Republic, China; 4 Key Laboratory of Analytical Chemistry for Biology and Medicine (Ministry of Education), College of Chemistry and Molecular Sciences, and State Key Laboratory of Virology, Wuhan University, Wuhan, 430072, People’s Republic China

**Keywords:** Gastric cancer, Prognosis, Tumor microenvironment, Lymph node ratio

## Abstract

**Background:**

Given the complexity of tumor microenvironment, no single marker from cancer cells could adequately predict the clinical outcomes of gastric cancer (GC). The objective of this study was to evaluate the prognostic role of combined features including conventional pathology, proteinase and immune data in GC.

**Methods:**

In addition to pathological studies, immunohistochemistry was used to assess membrane-type 1 matrix metalloproteinase (MT1-MMP) expression and CD11b + immunocytes density in three independent GC tissue microarrays containing 184 GC tissues. Separate and combined features were evaluated for their impact on overall survival (OS).

**Results:**

We found that traditional factors including tumor size, histological grade, lymph node status, serosa invasion and TNM stage were associated with OS (*P* < 0.05 for all). Moreover, statistically significant differences in OS were found among lymph node ratio (LNR) subgroups (*P* < 0.001), MT1-MMP subgroups (*P* = 0.015), and CD11b + immunocytes density subgroups (*P* = 0.031). Most importantly, combined feature (MT1-MMP positive, low CD11b + immunocytes density and high LNR) was found by multivariate analysis to be an independent prognostic factors for OS after excluding other confounding factors (HR = 3.818 [95%CI: 2.223-6.557], *P* < 0.001). In addition, this combined feature had better performance in predicting clinical outcomes after surgery long before recurrence had occurred (Area under the curve: 0.689 [95%CI: 0.609-0.768], *P* < 0.001).

**Conclusions:**

These findings indicate that better information on GC prognosis could be obtained from combined clinico-pathological factors, tumor cells and the tumor microenvironment.

## Background

Gastric cancer (GC) is the fourth most common cancer and the third cause of cancer death worldwide [1]. Considerable progress has been made in the early diagnosis of cancer, but there has not been a comparable advance in the accuracy of clinical outcome prediction [2]. Only the TNM classification of the International Union Against Cancer (UICC) or American Joint Committee on Cancer (AJCC) is used on a routine basis, which is the most important instrument to guide treatment strategy for GC patients [3]. However, GCs have significant heterogeneity in their biologic behaviors, and tumors of the same clinical stage often show differences in clinical course and treatment response [4]. Thus, identification of factors affecting invasion and metastasis, and establishment of biomarkers panels to predict malignant potential and to identify different risk groups are of a paramount importance.

Despite years of research and hundreds of reports on tumor markers in GC, the number of clinically useful markers is pitifully small [5]. Recently, a new prognostic tool of lymph node ratio (LNR), defined as the number of MLN divided by the number of lymph nodes retrieved, was proposed. It can improve the prognosis accuracy and reduce the stage migration when compared with the UICC (2002, 6th edition) staging system [6,7]. LNR has been considered as one of the best clinicopathologic variables for prediction of prognosis of gastric cancer after curative surgery in retrospective studies [8], which is the success in the optimization of traditional clinical markers. However, the discovery of other so-called promising markers in laboratory translates rather slowly into clinical applicability. One reason for this is the fact that cancer development and progression is determined by the co-evolution between tumor cells and tumor microenvironment rather than a single mutation [9]. Such co-evolution has been reported by many studies, all of which indicate that interactions between tumor cells and tumor stromal create a unique and dynamic microenvironment to affect tumor progression [10]. Therefore, screening new molecular factors from the complex tumor microenvironment (cellular and stromal components) represents another essential approach to identify prognostic factors.

MT1-MMP is known as a membrane-type matrix metalloproteinase, and MT1-MMP–mediated extracellular matrix (ECM) degradation by tumor cells or stromal cells is essential for cancer invasion through basement membranes and stromal interstitial matrix [11]. Although MT1-MMP has shown prognostic significance in several human cancers, such as breast cancer, pancreatic cancer and colorectal cancer [12,13], little is known about the accurate significance of MT1-MMP in GC patients. Major contributors to the tumor microenvironment are inflammation and inflammatory mediators [9]. It has been proposed that the immune contexture may influence the clinical outcome of the cancer patients, and the immune score would be important predictor comparable to TNM classification [14]. CD11b is expressed by a specific subset of myeloid cells, including CD34+ progenitors, monocytes, granulocytes, NK cells and activated T cells, accounting for the innate immunity [15,16]. The role of tumor immunogenicity and immunotherapies are being discussed [17]. Extending our knowledge of the complex role of immune cells in GC could ultimately pave the way for the long-awaited successful development of therapeutics.

Based on this understanding, we investigated prognostic role of cellular proteinase marker MT1-MMP and microenvironment immune marker CD11b + immunocytes in GC. Special attention was paid to the prognostic value of the combined features of MT1-MMP, CD11b + immunocytes density and LNR.

## Methods

### Patients and follow-up

The records of GC patients who underwent surgery with a curative intent at the Department of Oncology, Zhongnan Hospital of Wuhan University (Wuhan, China) between December 2002 and February 2011 were reviewed. Major demographic and clinico-pathological characteristics were available. No patients received neoadjuvant chemotherapy before surgery. TNM stage was determined according to the 7th edition UICC/AJCC TNM system [18]. By the most recent follow-up on May 31, 2012, the median follow-up was 59.5 (range: 16.8-102.3) months. A total of 108 (58.7%) patients died. Overall survival (OS) was defined as the interval from the date of surgery to GC-related death. Any recurrence in abdomino-pelvic cavity was defined as local-regional recurrence; and recurrence via blood flow was defined as distant metastasis, such as liver metastasis and lung metastasis. Written informed consent was obtained from the patients and the study protocol was approved by the ethics committee of Zhongnan Hospital of Wuhan University. The study was undertaken according to the ethical standards of the World Medical Association Declaration of Helsinki.

### Gastric cancer specimens and tissue microarrays

All hematoxylin and eosin (HE)-stained slides were examined by independent reviewers who were not aware of the clinical characteristics or clinical outcomes. Three tissue microarrays (TMAs) were constructed. Briefly, two cores were taken from each representative tumor tissue (1.5 mm in diameter for each core). Then, three TMAs sections with 184 tumor tissues were constructed (in collaboration with Shanghai Biochip Company Ltd., Shanghai, China).

### Immunohistochemistry and evaluation of immunohistochemical findings

The primary antibodies for MT1-MMP (sc101451, Santa Cruz, USA, dilution 1/100), CD11b + (ab52478, Abcam, UK, dilution 1/100), the corresponding horseradish peroxidase (HRP) conjugated secondary antibody (ab97265, ab97200, Abcam, UK, dilution 1/300), and diaminobenzidine (DAKO, Denmark) were obtained and validated for labeling.

The immunohistochemistry protocols were described previously [19]. In short, three TMAs sections were deparaffinised with xylene thenrehydrated through three changes of alcohol. Endogenous peroxidase activity was blocked by 0.3% hydrogen peroxide for 10 min. Antigen retrieval was performed by microwave treatment in 0.01 M Tris-EDTA buffer (pH 9.0) for 20 min. Slides were incubated for 1 h with the primary antibodies for MT1-MMP (sc101451, Santa Cruz, USA, dilution 1/100), CD11b + (ab52478, Abcam, UK, dilution 1/100). After washing with Tris-buffered saline (TBS, pH 7.4), sections were incubated with b the corresponding horseradish peroxidase (HRP) conjugated secondary antibody (ab97265, ab97200, Abcam, UK, dilution 1/300) for a further 50 min. Antigen–antibody reaction was visualised with 3,30-diaminobenzidine (DAKO, Denmark). After counterstaining with haematoxylin, sections were dehydrated through ascending alcohols to xylene and mounted. Positive staining controls were performed in parallel with paraffin sections of normal human tonsil. Negative control was performed by omitting the primary antibody.

The slides were examined under Olympus BX51 fluorescence microscope equipped with an Olympus DP72 camera (Olympus Optical Co., Ltd., Tokyo, Japan). Panorama of each labeled core was obtained based on 4 photographs under low-power magnification (×100) with high resolution by DP-BSW software (Olympus Optical Co., Ltd., Tokyo, Japan). Identical settings were used for every photograph, so as to minimize the selection bias. The MT1-MMP expression and CD11b + immunocytes density were digitally evaluated by Image-Pro Plus v6.2 software (Media Cybernetics Inc, Bethesda, MD). To keep results reliable and comparable, a uniform setting for all the slides was applied for the reading of each antibody staining. Integrated optical density (IOD) of all the positive staining of MT1-MMP in each photograph was measured, and its ratio to total area of each photograph was calculated as MT1-MMP expression. CD11b^+^ immunocytes density was recorded as the number of positive cells per tissue surface unit in square millimeters. Cut points for MT1-MMP density was explored by “the best cut-off approach by log-rank test” [20]. And the 25th percentile value was defined as the cut-off point to determine the MT1-MMP negative and positive expression subgroups in this study. For the CD11b + immunocytes density, the cut-off point for the definition of subgroups (low and high CD11b + immunocytes density) was the median value.

### Statistical analysis

Statistical analyses were performed with SPSS software (version 18.0, SPSS Institute, Chicago, IL). The Pearson χ^2^ test or Fisher’s exact test was used to compare qualitative variables. Kaplan-Meier analysis was used for survival analysis and significance among patients’ subgroups was calculated by log rank test. The Cox regression model was used to perform multivariate analysis. Logistic regression was used to assess the influence of binary factors. Receiver operating characteristic curve (ROC) analysis was used to determine the predictive value of the parameters. Two sided *P* < 0.05 was considered as statistically significant.

## Results

### Major clinico-pathological features and immunohistochemical findings

Among the 184 cases included in this study were 132 (71.7%) males and 52 (28.3%) females, ranging in age from 25 to 85 yr (Mean ± SD: 57.9 ± 12.9 yr). The main demographic and clinico-pathological characteristics were presented in Table 1.

**Table 1:** **Patients**’ **demographics and clinico**-**pathological characteristics**

**Items**	**Value**
Age (M ± SD, yrs)	57.9 ± 12.9
Gender	
Male (%)	132 (71.7)
Female (%)	52 (28.3)
Tumor location	
Distal stomach (%)	98 (53.3)
Non-distal stomach (%)	86 (46.7)
Pathological types	
Adenocarcinoma	
Histological grade 1/2	55 (29.9)
Well/Moderately differentiated adenocarcinoma (%)	55 (29.9)
Histological grade 3/4	124 (67.4)
Low/Undifferentiated adenocarcinoma (%)	102 (55.4)
Mucinous adenocarcinoma/signet-ring cell carcinoma	22 (12.0)
Others ^a^ (%)	5 (2.7)
Serosa invasion	
No (T0, T1, T2) (%)	27 (14.7)
Yes (T3, T4) (%)	157 (85.3)
Lymph node metastasis	
No (N0) (%)	53 (28.8)
Yes (N1, N2, N3) (%)	131 (71.2)
LNR, Median (Range)	0.33 (0-1)
Distant Metastasis	
M0 (%)	177 (96.2)
M1 (%)	7 (3.8)
TNM stages	
Early (Stages I, II) (%)	58 (31.5)
Advanced (Stages III, IV) (%)	126 (68.5)
Surgery	
Subtotal resection (%)	159 (86.4)
Non-Subtotal resection (%)	25 (13.6)
Chemotherapy	
No (%)	35 (19.0)
Yes (%)	149 (81.0)
Recurrence	
No (%)	124 (67.4)
Yes (%)	60 (32.6)
Recurrence location ^b^	
Local-regional	37 (61.7)
Distant	23 (38.3)
Clinical status at the end of the follow up	
Live and without recurrence	72 (39.1)
Dead or alive with recurrence	112 (60.9)
MT1-MMP expression	
Negative	46 (33.3)
Positive	138 (66.7)
CD11b + immunocytes (cells/mm^2^)	
Median (range)	257 (4-2101)

^a^ Others included adenosquamous carcinoma in 3 (1.62%) cases, untypical carcinoid in 1 (0.54%) case, and neuroendocrine carcinoma in 1 (0.54%) case.^b^ 60 recurrent cases were evaluated.

**Figure 1:**
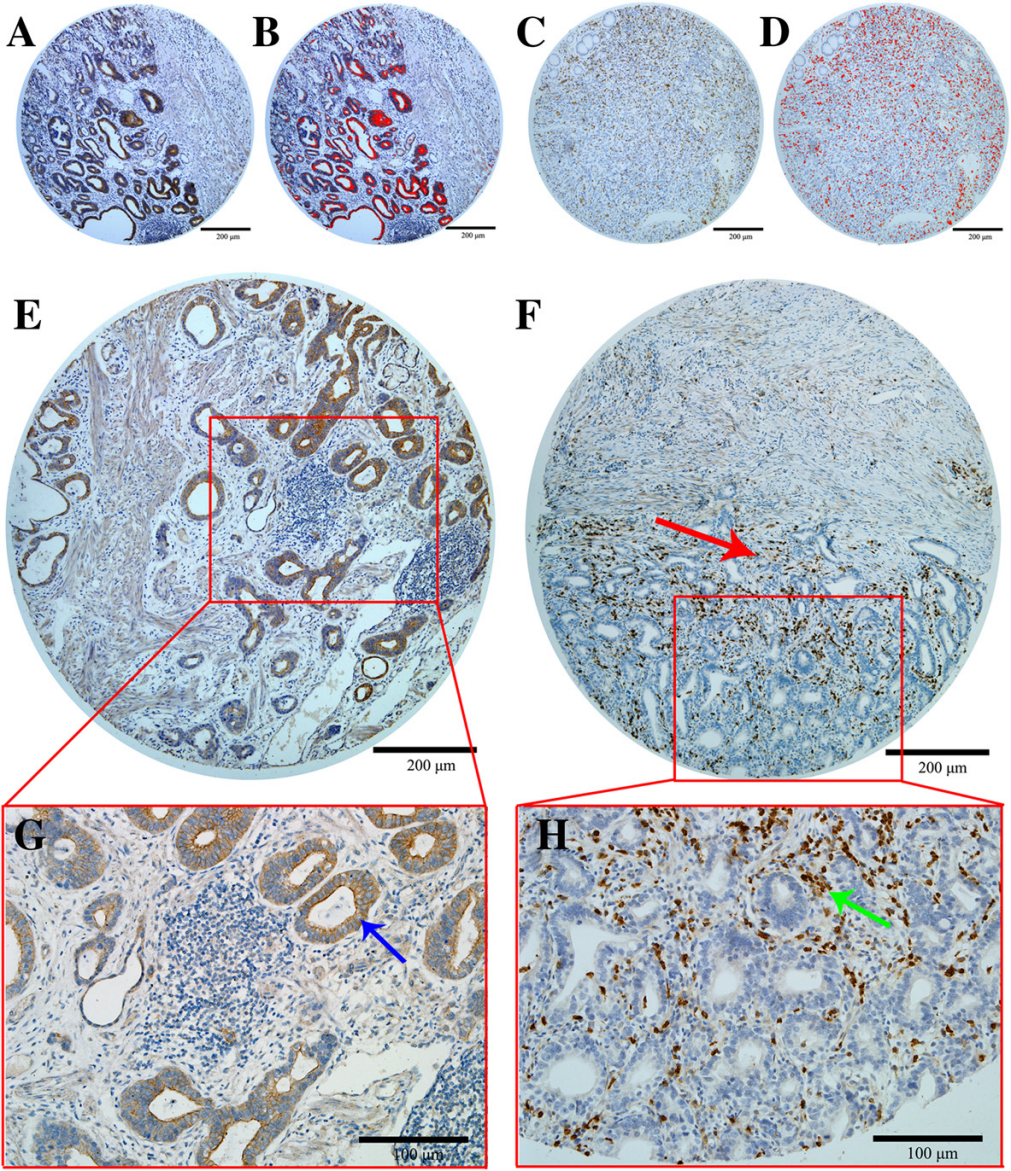
**Immunohistological findings in TMAs.** (**A**-**D**) Representative example of MT1-MMP (**A**), CD11b + immunocytes (**C**) staining of a GC tissue microarray, and the corresponding digital image analyzed with the image software, with MT1-MMP (**B**) and CD11b + immunocytes (**D**) represented in red. (**E**,**G**) MT1-MMP staining was mainly in the cytoplasm or on the cell membrane of tumor cells (Blue arrow), and most of the stroma cells had negative staining, although sporadic positive staining on these cells was also observed (**F**,**H**). CD11b staining showed that CD11b + immunocytes mainly infiltrated into the juncture of cancer nest and stroma, especially at the invasive front (Red and Green arrows).

Immunohistochemistry was performed in all GC TMAs and the result of each specimen was obtained for images-based digital analysis (Figure 1A-1D). MT1-MMP staining was mainly in the cytoplasm or on the cell membrane of tumor cells. Most of the stromal cells were negative, although sporadic positive staining on these cells was also observed (Figure 1E, 1G). CD11b + immunocytes mainly infiltrated into the juncture of cancer nest and stromal (Figure 1F, 1H). The level of MT1-MMP density and CD11b + immunocytes density were presented in Table 1. The 25th percentile of MT1-MMP density was 0.00186 and 138 (75.0%) patients were documented as MT1-MMP positive according to the abovementioned classification criteria. The median value of CD11b + immunocytes were 257 cells per tissue surface unit in square millimeter.

### Relationship between MT1-MMP expression, CD11b + immunocytes density and clinico-pathological features

There were 77 (55.8%) and 15 (32.6%) patients with high CD11b + immunocytes density in MT1-MMP positive (*n* = 138) and negative (*n* = 46) subgroups, respectively (*P* = 0.006). The likelihood of MT1-MMP expression was decreased significantly for low CD11b + immunocytes density subgroup (odds ratio (OR) = 0.383 [95%CI: 0.190-0.774], *P* = 0.007, Logistic regression). The MT1-MMP expression and CD11b + immunocytes density were correlated with the clinico-pathological features as summarized in Table 2. MT1-MMP expression was correlated with small tumor size (*P* = 0.007), tumor at distal stomach (*P* = 0.027) and increased recurrence risk (*P* = 0.001), but not correlated with age, gender, histological grade, serosa invasion, lymph node status, TNM stage and sites of tumor recurrence (*P* > 0.05 for all). CD11b + immunocytes density was correlated with lymph node status (*P* = 0.015), LNR (*P* = 0.018) and TNM stage (*P* = 0.011), but not correlated with age, gender, tumor size, tumor location, histological grade, serosa invasion and recurrence risk (*P* > 0.05 for all).

**Table 2:** **The relationship between MT1**-**MMP**, **CD11b** + **immunocytes density and cinico**-**pathological features**

**Variables**	**MT1-MMP expression**			**CD11b** **+** **immunocytes density**		
	**Negative** (*n* = 46)	**Positive** (***n*** = 138)	***P***	**Low** (*n* = 92)	**High** (*n* = 92)	***P***
	*N* (%)	*N* (%)		*N* (%)	*N* (%)	
Age (yrs)						
<60	26 (56.5)	20 (43.5)	0.669	43 (43.4)	56 (56.6)	0.055
≥60	73 (52.9)	65 (47.1)		49 (57.6)	36 (42.4)	
Gender						
Male	33 (25.0)	99 (75.0)	1.000	66 (50.0)	66 (50.0)	1.000
Female	13 (25.0)	39 (75.0)		26 (50.0)	26 (50.0)	
Tumor size (cm^2^) ^a^						
<16	12 (16.4)	61 (83.6)	0.007	35 (47.9)	38 (52.1)	0.404
≥16	31 (35.2)	57 (64.8)		48 (54.5)	40 (45.5)	
Location						
Distal	18 (18.4)	80 (81.6)	0.027	51 (52.0)	47 (48.0)	0.555
Non-distal	28 (32.6)	58 (67.4)		41 (47.7)	45 (52.3)	
Histological grade ^b^						
1/2	13 (23.6)	42 (76.4)	0.845	24 (43.6)	31(56.4)	0.376
3/4	31 (25.0)	93 (75.0)		63 (50.8)	61 (49.2)	
Lymph node metastasis						
Yes	31 (23.7)	100 (76.3)	0.511	73 (55.7)	58 (44.3)	0.015
No	15 (28.3)	38 (71.7)		19 (35.8)	34 (64.2)	
LNR ^c^						
≤0.33	27 (28.7)	67 (71.3)	0.233	39 (41.5)	55 (58.5)	0.018
>0.33	19 (21.1)	71 (78.9)		53 (58.9)	37 (41.1)	
Serosa invasion (T stage)						
T1-2	4 (14.8)	23 (85.2)	0.186	10 (37.0)	17 (63.0)	0.145
T3-4	42 (26.8)	115 (73.2)		82 (52.2)	75 (47.8)	
TNM staging						
Early (I, II)	13 (22.4)	45 (77.6)	0.583	21 (36.2)	37 (63.8)	0.011
Advanced (III, IV)	33 (26.2)	93 (73.8)		71 (56.3)	55 (43.7)	
Recurrence						
Yes	9 (15.0)	51 (85.0)	0.029	30 (50.0)	30 (50.0)	1.000
No	37 (29.8)	87 (70.2)		62 (50.0)	62 (50.0)	
Recurrence location ^d^						
Local-regional	7 (18.9)	30 (81.1)	0.460	20 (54.1)	17 (45.9)	0.426
Distant	2 (8.7)	21 (91.3)		10 (43.5)	13 (56.5)	

^a^ Analysis was performed based on 161 patients with complete data of tumor size (TS).^b^ Analysis was performed based on 179 patients with gastric adenocarcinoma.^c^ The median of lymph node ratio (LNR) was 0.33 (6 cases), and GC cases were different in distinct subgroups. ^d^ 60 recurrent cases were included.

### Survival analysis

**Figure 2:**
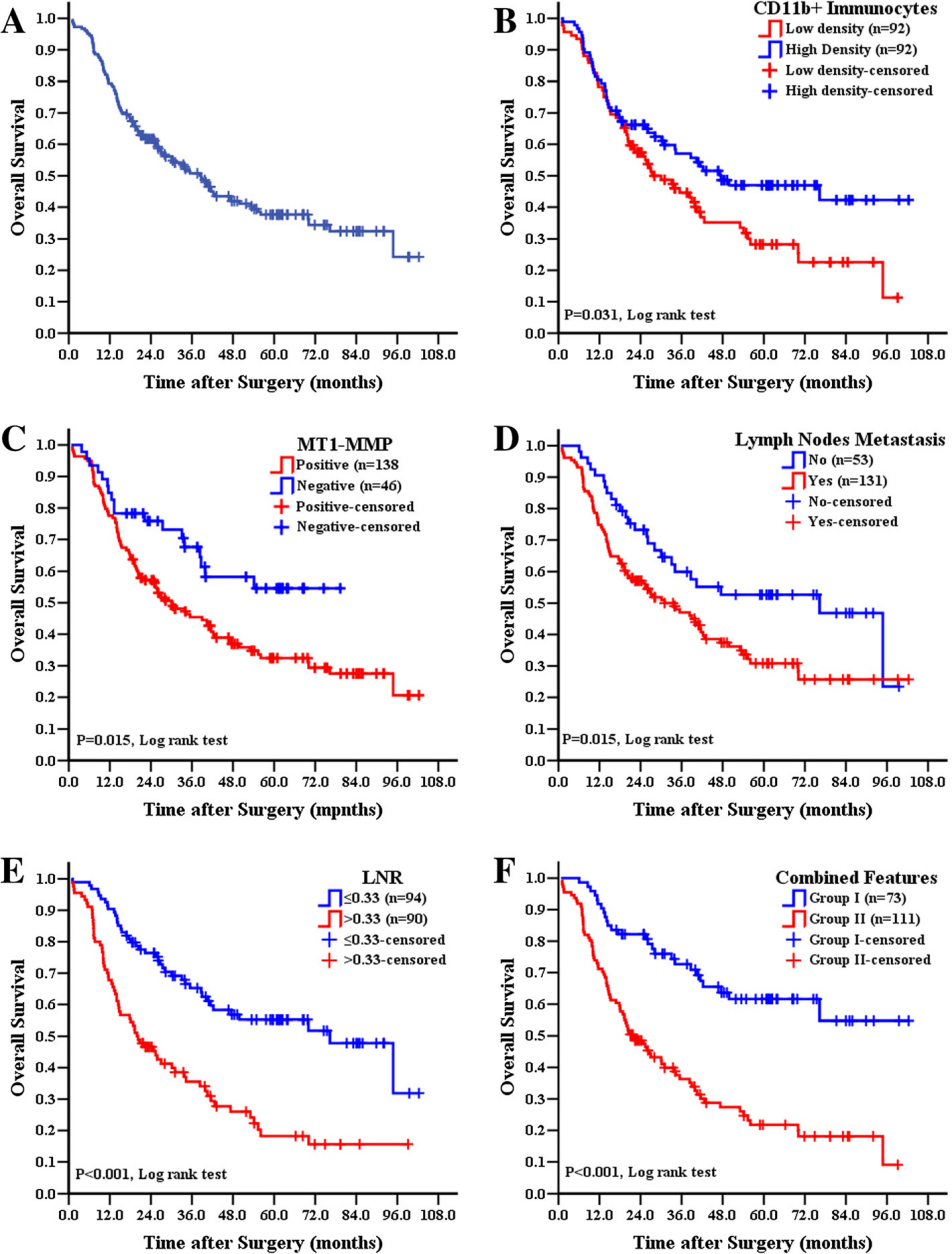
**Cumulative OS of GC patients.** (**A**) OS of 184 GC patients. (**B**) Low CD11b + immunocytes density was related to poor OS. (**C**) Patients in MT1-MMP positive group were at higher risk for death. (**D**, **E**) Both lymph nodes status and LNR were correlated with poor OS, but LNR could better differentiate patient subgroups. (**F**) Combined Features could indicate OS of GC patients more accurately.

For 184 cases, the median OS was 26.8 (range 0.8-102.3) months, and the 1-, 3-, and 5-yr survival rate was 79.3%, 50.5% and 37.4%, respectively (Figure 2A). As expected, those traditional factors were associated with GC patients’ OS, such as tumor size, histological grade, lymph node status, serosa invasion and TNM stage (*P* < 0.05 for all). In addition, both high CD11b + immunocytes density and MT1-MMP negative expression were correlated with better OS (*P* value was 0.031 and 0.015, respectively.) (Figure 2B, 2C). The discrimination ability in OS was increased in LNR subgroups (*P* < 0.001) compared to lymph nodes status subgroups (*P* = 0.015) (Figure 2D, 2E ), and ROC analysis showed that, in terms of predicting risk of death, the classification by LNR (Area under the curve: 0.659 [95%CI: 0.579-0.739], *P* < 0.001) had better performance than positive lymph nodes number (Areas under the curve: 0.568 [95%CI: 0.484-0.653], *P* = 0.114). All results indicated that MT1-MMP positive, low CD11b + immunocytes density and high LNR were risk factors related to poor prognosis (Figure 2, Table 3).

**Table 3:** Analyses of factors regarding OS

**Variables**	**N**	**N of death (%)**	**Median OS (Range)**	**5-year survival rate (%)**	**Log-rank test χ**^**2 **^**value**	***P***
Age (yrs)						
<60	99	52 (52.5)	30.1 (1.5-99.5)	45.1	3.584	0.058
≥60	85	56 (65.9)	24.8 (0.8-102.3)	28.6		
Gender						
Male	132	74 (56.1)	30.3 (0.8-102.3)	40.0	2.114	0.146
Female	52	34 (65.4)	25.1 (1.3-94.8)	30.3		
Tumor size (cm^2^) ^a^						
<16	73	35 (47.9)	41.6 (1.1-99.5)	52.9	7.882	0.005
≥16	88	57 (64.8)	24.2 (0.8-102.3)	29.2		
Histological grade ^b^						
1/2	55	88 (56.1)	30.1 (1.1-102.3)	54.2	13.412	<0.001
3/4	124	20 (74.1)	16.8 (0.8-74.9)	30.1		
Lymph nodes metastasis						
Yes	131	83 (63.4)	24.8 (0.8-102.3)	52.9	5.895	0.015
No	53	25 (47.2)	34.1 (6.1-99.5)	30.5		
Serosa invasion (T stage)						
T1-2	27	7 (25.9)	58.9 (20.2-99.5)	74.9	14.742	<0.001
T3-4	157	101 (64.3)	24.8 (0.8-102.3)	30.8		
TNM						
Early (I, II)	58	26 (44.8)	35.8 (6.1-99.5)	54.0	9.823	0.002
Advanced	126	82 (65.1)	22.8 (0.8-102.3)	29.2		
(III, IV)						
Surgery						
SR	159	89 (56.0)	30.0 (0.8-102.3)	39.8	5.902	0.015
TR/CR	25	19 (76.0)	18.0 (3.8-89.9)	21.8		
Chemotherapy						
Yes	149	87 (49.7)	33.5 (5.3-99.5)	35.9	0.016	0.900
No	35	21 (60.0)	28.4 (0.8-102.3)	40.0		
LNR ^c^						
≤0.33	94	41 (43.6)	38.8 (1.1-102.3)	55.1	24.881	<0.001
>0.33	90	67 (74.4)	20.1 (0.8-99.1)	18.3		
MT1-MMP						
Negative	46	18 (39.1)	35.7 (3.8-79.3)	54.0	5.869	0.015
Positive	138	90 (65.2)	25.4 (0.8-102.3)	32.2		
CD11b + immunocytes density						
Low	92	62 (67.4)	25.1 (0.8-99.1)	27.6	4.655	0.031
High	92	46 (50.0)	30.7 (1.3-102.3)	46.7		
Combined features (MT1-MMP density, CD11b + immunocytes density and LNR)						
Group I	73	26 (35.6)	38.7 (6.1-94.1)	61.4	28.173	<0.001
Group II	111	82 (73.9)	18.1 (0.8-90.9)	21.1		

^a^ Analysis was performed based on 161 patients with complete data of tumor size. ^b^ Analysis was performed based on 179 patients with gastric adenocarcinoma. ^c^ The median of LNR was 0.33 (6 cases), and GC cases were different in distinct subgroups.OS: overall survival; SR: subtotal resection; TR: total resection; CR: combined resection; LNR: lymph node ratio.

As we proposed above, combined features based on the number of risk factors were explored to improve prediction of GC prognosis (Table 3). Thus, patients were divided into two subgroups according to the number of risk features: group I, less than two risk factors were observed; and group II, two or three risk factors were observed. Combined analysis showed that the OS of patients in group II was significantly shorter than patients in group I (*P* < 0.001) (Figure 2F).

### Multivariate analysis and ROC analysis

In univariate analysis, traditional clinico-pathological features (such as tumor size, T stage, TNM stage, surgery methods, and recurrence status), MT1-MMP expression, CD11b + immunocytes density and LNR were associated with OS. Furthermore, the death risk in combined group II increased significantly (*P* < 0.001).

Factors showing significance by univariate analysis were integrated into multivariate Cox proportional hazards analysis. In this study, LNR, MT1-MMP, CD11b and combined features were highly correlated. Therefore, two separate multivariate models were generated to avoid the multicollinearity among those variables. LNR, MT1-MMP expression and CD11b + immunocytes density were independent prognostic factors for OS after excluding other confounding factors (*P* < 0.05 for all) Moreover, the combined features were independent prognostic factor, too (HR = 3.818 [95%CI: 2.223-6.557], *P* < 0.001) (Table 4).

**Table 4:** Multivariate analyses of factors associated with OS

**Factors**	**OS**		
	**HR**	**95% CI**	***P***
**Model 1**			
Tumor size: <16 cm^2^*vs* ≥16 cm^2^	1.821	1.168-2.839	0.008
Serosa invasion (T stage): T1-2 *vs* T3-4	3.232	1.440-7.251	0.004
Lymoh nodes metastasis: No *vs* Yes	1.012	0.610-1.679	0.964
Surgery types: SR *vs* TR/CR	2.030	1.128-3.653	0.018
LNR: low (≤0.33) vs High (>0.33)	1.957	1.233-3.108	0.004
MT1-MMP: Negative *vs* Positive	2.596	1.496-4.506	0.001
CD11b + immunocytes density: High *vs* Low	1.838	1.183-2.855	0.007
**Model 2**			
Tumor size: <16 cm^2^*vs* ≥16 cm^2^	1.734	1.120 - 2.686	0.014
Serosa invasion (T stage): T1-2 *vs* T3-4	3.013	1.360 - 6.674	0.007
Lymoh nodes metastasis: No *vs* Yes	0.696	0.404 - 1.200	0.192
Surgery types: SR *vs* TR/CR	2.023	1.134 - 3.606	0.017
Combined features: Group I *vs* Group II	3.818	2.223 - 6.557	<0.001

OS: overall survival; HR: hazard ratio; NA: not adopted; SR: subtotal resection; TR: total resection; CR: combined resection; LNR: lymph node ratio.

**Figure 3:**
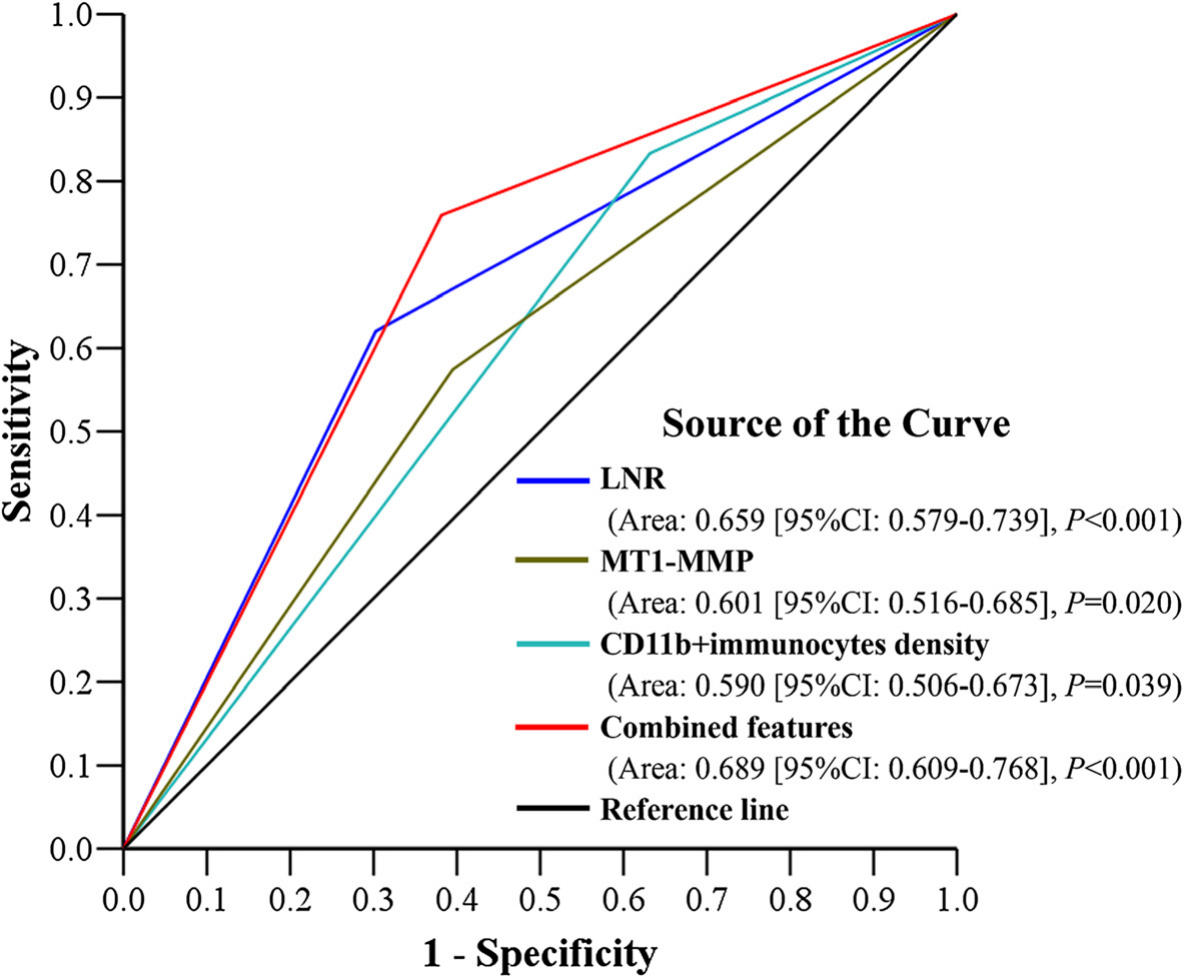
**ROC analysis of the predictive value for death.** Among the 4 independent prognostic factors, the area under the curve of the new combined feature was the largest one. The combined feature could have better prognostic performance in GC patients.

ROC analysis was implemented to further evaluate the prognostic performance of the four independent factors in this study. Combined features would be better to predict the clinical outcomes of GC patients compared to other factors (Area under the curve: 0.689 [95%CI: 0.609-0.768], *P* < 0.001) (Figure 3).

## Discussion

We have proposed a combined analysis of integrated tumor stromal features as a useful strategy to evaluate cancer progression and patient survival in GC based on our studies focused on the co-evolution of tumor cells and tumor microenvironment and [19,21,22]. This study was designed to explore the feasibility of this combined strategy. In addition, an improved automation method to analyze the digitalized images was used to ensure both reproducibility and good performance in this study. As Fridman [23] suggested, such methods would pave the way to better understanding the complex tumor microenvironment, as well as to the routine evaluation of parameters for clinical management of cancer patients. Herein, 184 GC cases were included to evaluate the prognostic values of optimized conventional pathological prognostic factors, cellular molecular factors, immune factors and the combined features. This is the essential step towards establishing a workable prognostic system integrating both clinico-pathological, tumor and stromal features in our series studies [19,21,22].

Of 184 cases, the demographics and clinico-pathological characteristics are similar to those reported in other large series of GC population [24]. Our results showed that the expression of MT1-MMP was frequently correlated with increased recurrence risk, but the difference in relapse location was not statistically significant. These results were similar to previous report [25]. MT1-MMP plays important role in degrading types I and IV collagens to facilitate cancer cells spreading. In addition, MT1-MMP can promote angiogenesis and micrometastasis via vascular route [26].

With regard to immune cells, the nature, density and location are important parameters to comprehensively evaluate the *in situ* immune reaction and the specific role in cancer progression. In this study, CD11b + immunocytes were mostly located at the invasive front. The difference in CD11b + immunocytes density was statistically significant between lymph nodes metastasis and non-metastais subgroups. Furthermore, the CD11b + immunocytes density was higher in early than advanced GC patients, similar to the reports by Sconocchia et al. and Ladoire et al. [27,28]. Hence, we hypothesized that CD11b + immunocytes could prevent the lymph nodes metastasis by active immunosurveillance process.

The prognostic value of traditional clinicopathological prognostic factors has been validated [29,30]. Interestingly, some studies reported that the LNR was a better predictor of patient outcome than lymph nodes status only. LNR may be an alternative stratification in cases where few nodes are retrieved [3,8]. LNR has also been adopted by the Japanese Gastric Cancer Association (JGCA) [31].

Researches focused on molecular factors for cancer progrosis have attracted increasing attention [32,33]. In this study, MT1-MMP expression and CD11b + immunocytes density were independent prognostic factors, which partly validated others’ conclusions about MT1-MMP and CD11b + immunocytes. Kanazawa et al. [34] reported that MT1-MMP expression could be considered as a useful independent predictor of outcomes in colorectal cancer patients. The results presented by Mahmoud et al. [35] confirmed the presence of efficient immunologic antitumor defense mechanisms in human breast cancer. It is proposed that immune score would identify a population of patients who would derive substantial benefit from further stimulating their immune response [36]. Several studies have also provided evidence of immune criteria to predict which tumors have a high risk of death [35,37].

Given the fact that tumor biology is often dictated by several essential cellular and microenvironmental alterations, it may be naive to think that single factor would be enough as prognostic factors in cancer [38]. Solutions are now being explored by analyzing multiple factors with tissue microarrays, which has been emerged as an essential tool in the discovery and validation of tissue biomarkers [39]. To our knowledge, combined analysis is a promising method to translate experimental results into clinical application [40]. Based on our results and current knowledge in cancer progression, we proposed a new prognostic model that combines pathological, cellular and molecular features. This study showed that the combined features were independent prognostic factors for OS. The death risk of GC patients in group II was increased by 200% and this combined features would better predict GC patients’ outcomes.

The development of tumor biomarkers ready for clinical use is complex, and a useful prognostic marker must be a proven independent, significant factor, that is easy to determine and interpret and has therapeutic impact [41]. Although the combined features described herein could address these conditions, the promising results are based on retrospective analysis, which is the limitation of this study. Prospective randomized clinical trials to evaluate the clinical utility of a prognostic or predictive biomarker are the gold standard, but such trials are costly and difficult to implement, and more efficient indirect “retrospective analysis” using archived specimens would be an alternative method for a long time [42].

## Conclusions

Our study provides evidence for the value of the combined features to predict OS in GC patients. The combined features of positive MT1-MMP, low CD11b + immunocytes density and high LNR may be used as useful prognostic factor in clinical circumstance in future. Further studies with larger sample size could help gain deeper insights into the role of combined features.

## Competing interests

The authors declare that they have no competing interests.

## Authors’ contributions

PCW selects the research topic, conducts the pathological examination, statistical analysis and writes manuscript. WLW, FM and YGF conduct the pathological examination. LY and PDW conceive the study project, organizes the whole study process, provides financial support, and finalizes the manuscript. All authors have read and approved the final manuscript.
